# An overview on the management of ankle fractures in elderly patients aged 65 and over: a scoping review

**DOI:** 10.1007/s00590-025-04512-0

**Published:** 2025-09-13

**Authors:** Gang Liu, Zhijian Sun, Minghui Yang, Xinbao Wu, Peter V. Giannoudis

**Affiliations:** 1 Present Address: Department of Orthopaedics Trauma, Beijing Jishuitan Hospital, Capital Medical University, Beijing, China; 2 Academic Department of Trauma and Orthopaedics, University of Leeds, Leeds, United Kingdom; 3 NIHR Leeds Biomedical Research Center, Chapel Allerton Hospital, Leeds, United Kingdom

**Keywords:** Aged 65 and over, Ankle fracture, Management, Complications

## Abstract

**Purpose:**

Ankle fractures are increasingly common in the elderly and present unique challenges due to osteoporosis, comorbidities, and frailty. Management remains controversial, with surgical fixation generally favored, though the optimal approach is debated. This scoping review aimed to map current evidence on the management of ankle fractures in patients aged 65 and over, highlighting treatment strategies, outcomes, and gaps in knowledge.

**Methods:**

A scoping review was conducted in accordance with The Preferred Reporting Items for Systematic Reviews and Meta-Analyses Extension for Scoping Reviews (PRISMA-ScR). We systematically searched PubMed, Embase, and Cochrane Library from 2010 to 2024. Eligible studies reporting treatment outcomes in elderly patients were included. Data were charted and summarized narratively; pooled descriptive analyses were presented to illustrate trends, consistent with PRISMA-ScR methodology.

**Results:**

A total of 4783 articles were identified, of which 32 met the inclusion criteria. Of the 32 studies analyzed, 24 were conducted in Europe, 5 in the United States, and 1 each in China, Korea, and New Zealand. Study designs comprised 27 retrospective observational studies, 3 randomized controlled trials, one prospective case series and one ambispective cohort study. Open reduction and internal fixation remain the standard surgical method, while alternatives such as tibiotalocalcaneal nailing and fibular intramedullary nailing are increasingly reported. Overall functional recovery was satisfactory, but outcomes varied by comorbidity burden, fracture pattern, and rehabilitation strategy. Complication rates were substantial, with wound problems, fixation failure, and infections most frequent. Mortality ranged from 10 to 27%, with higher rates in nonoperative and frail populations. Evidence on weight-bearing protocols and long-term outcomes remains inconsistent.

**Conclusions:**

Current evidence supports surgery as the preferred option for most elderly ankle fractures, though individualized, multidisciplinary care is essential. Research gaps include standardized fixation protocols, comorbidity-adjusted pathways, and long-term functional outcomes. High-quality prospective studies are needed to refine clinical recommendations.

## Introduction

Ankle fractures are common injuries in the elderly because of osteoporosis, making them the third most common fragility fracture [1]. The incidence and prevalence of ankle fractures in elderly patients are expected to increase significantly as the global population aged ≥ 65 years grows, projected to reach 16% of the world population by 2050; in China alone, the population aged ≥ 65 years is expected to more than double from 172 million (12.0%) to 366 million (26.0%) by 2050 [2]. As a result, geriatric ankle fractures will pose a significant burden on society and healthcare systems.

Managing ankle fractures in elderly patients is challenging because they often have thin, fragile skin, multiple comorbidities, poor peripheral blood supply, and osteoporosis, all of which increase the risk of complications and mortality [3, 4]. Reported rate of complications for surgically repaired ankle fractures in patients aged ≥ 65 years have been as high as 30–40% [5, 6], whereas in younger patients postoperative frequency of complications are around 4.27% or lower [7]. Geriatric ankle fractures are also associated with a significant loss of independence; one study reported that up to 60% of these patients did not return to independent living. These injuries additionally have an elevated one-year mortality rate, approximately 12% for elderly ankle fracture patients [4]. However, mortality is even higher for open ankle fractures, reaching up to 27.3% [3].

In frail elderly patients, the primary treatment goal after a fracture is rapid mobilization. Although best-practice guidelines for managing hip fractures in this population are well established, the optimal treatment approach for geriatric ankle fractures remains under debate [4]. Poor mechanical properties of osteoporotic bone in osteoporotic patients can result in more complex ankle fracture patterns, making fracture fixation extremely difficult. Unlike hip fractures, ankle fractures have a smaller soft-tissue envelope around the joint that increases the risk of wound complications, surgical site infections, and malunion [8]. Although some consensus guidelines exist on certain aspects of perioperative management for elderly ankle fracture patients, considerable differences of opinion remain regarding care pathways, optimal fixation methods, and rehabilitation strategies [9, 10]. This reflects a relative lack of evidence in the field and has led to variability in treatment plans.

To address the question of how to best manage geriatric ankle fractures, this scoping review was conducted to systematically summarize the current evidence on ankle fracture management in patients aged ≥ 65 years, identify research gaps, and provide insights to guide future clinical decision-making and research.

## Methods

### Protocol and registration

This scoping review was conducted in accordance with the Preferred Reporting Items for Systematic Reviews and Meta-Analyses Extension for Scoping Reviews (PRISMA-ScR) [11, 12]. A protocol for this scoping review was prospectively registered on the Open Science Framework (10.17605/OSF.IO/65SXU).

### Eligibility criteria

The predefined eligibility criteria were developed based on the PICOTS (Population, Intervention, Comparison, and Outcome) framework, (Table 1) [13]. Eligible studies included randomized controlled trials, controlled trials, or observational studies (including case series and case–control studies) with at least 20 patients, published between January 2010 and January 2025. Systematic reviews, expert opinions, case reports, conference abstracts and letters were excluded.

**Table 1:** The inclusion and exclusion criteria

	Inclusion criteria	Exclusion criteria
Population(s)	Patients who were 65 years or older sustaining an ankle fracture	Multi trauma patients, pathologic fractures, distal tibial fractures and pilon-type fractures
Interventions	Studies investigating management of ankle fractures	Studies that do not include any management of ankle fractures
Comparisons	No restriction	No restriction
Outcomes	Studies had at least one of the following clinical outcomes, including functional outcome, complications, mortality and bony union	Studies that do not report any clinical outcomes
Time	Published between 1 January 2010 and 20 January 2025	–
Study design	Randomized controlled trials, controlled trials or observational studies, including case series and case–control studies including 20 patients at least	Review papers and systematic reviews, expert opinions, case reports, meeting and conference abstracts, letters
Other	Studies published in English	Abstract or conference abstract only

### Information sources

To ensure a comprehensive search of the published research literature, the following electronic databases were searched on January 2, 2025: PubMed/MEDLINE, Cochrane Library, Embase and Web of Science. In addition, we examined reference lists of selected articles to identify any studies missed in the initial search. We also conducted a targeted grey literature search of clinical trial registries.

### Search strategy

The search strategy was developed by the research team, with input from a senior librarian who reviewed the strategy. The search strategy for PubMed is presented in (Table 2). The search strategy, including all identified keywords and index terms, was adapted for each database and the grey literature search. No language filters were applied in the initial search; non-English articles were instead removed at the full-text screening stage. The literature search was restricted to publications from January 2010 through January 2, 2025.

**Table 2:** PubMed search query

1	Aged [MeSH terms]
2	Elderly [Title/abstract]
3	Geriatric [Title/abstract]
4	Fragility [Title/abstract]
5	Osteoporotic [Title/abstract]
6	ankle fractures [MeSH terms]
7	Ankle Fracture [Title/abstract]
8	Malleolus Fractures [Title/abstract]
9	Bimalleolar Fracture [Title/abstract]
10	Trimalleolar Fractures [Title/abstract]
11	#1 or #2 or #3 or #4 or #5
12	#6 or #7 or #8 or #9 or #10
13	#11 and #12

### Selection process

All articles from the database searches were imported into EndNote X8, and duplicates were removed. Two reviewers (LG and SJZ) independently screened titles and abstracts, followed by full-text screening for potentially relevant articles. Study inclusion was determined based on the prespecified inclusion and exclusion criteria. To avoid duplicate populations, we examined all included studies for overlap in institution, study period, and patient characteristics. When two or more studies were from the same center and had overlapping timeframes, we included only the most complete or the most recent publication. Any disagreements were resolved through discussion, and a third reviewer (PVG) was consulted in cases of continued uncertainty to reach a consensus.

### Data charting process

A standardized data extraction form was developed in Microsoft Excel to ensure consistent data collection. Both reviewers were trained to use the extraction form. One reviewer (LG) initially extracted the data, and the other (SJZ) verified it. The data extraction form was refined iteratively as needed. Any disagreements were resolved through discussion or, if needed, by consultation with a third reviewer (PVG).

### Data items

Data extracted from each study included:

1. General data (authors, publication year, country),
2. Methodological data (study design, sample size),
3. Fracture characteristics (e.g. open or closed, fracture type),
4. Intervention characteristics (e.g. type of treatments, surgical or non-surgical, fixation method, weight-bearing protocol),
5. Clinical outcomes: quality of life, functional outcome, postoperative complications, mortality.

The Template for Intervention Description and Replication (TIDieR) checklist was used to guide the extraction of intervention details [14]. No formal risk-of-bias assessment was conducted in this scoping review.

### Synthesis of results

The included studies were synthesized using descriptive methods. Findings were summarized to describe the nature and extent of the evidence for each outcome of interest and to identify gaps in the current literature. In addition, descriptive pooled analyses were performed for selected outcomes. Forest plots were generated to visualize the distribution of results across studies. These analyses were conducted using RevMan version 5.4 (Cochrane Collaboration), applying a random-effects model to account for between-study heterogeneity. Effect estimates were expressed as pooled means with 95% confidence intervals. These pooled values are descriptive in nature and were not intended as inferential meta-analysis results.

## Results

### Selection of sources of evidence

**Fig. 1:**
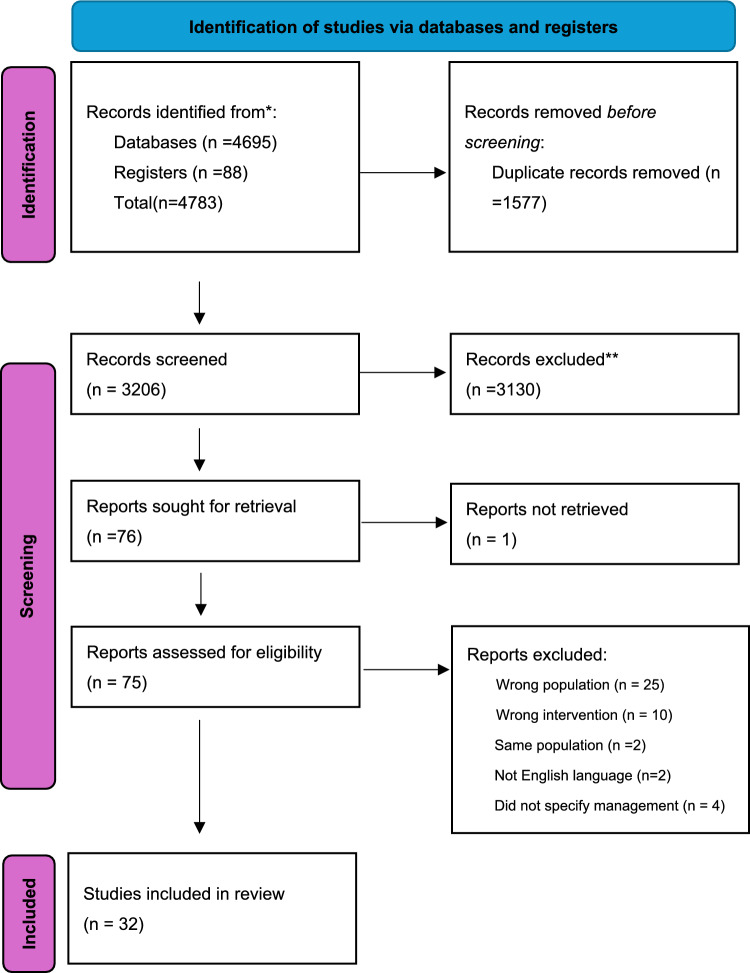
PRISMA flow diagram for study selection

A PRISMA flow diagram summarizing the search results is presented in (Fig. 1) [15]. The initial database search yielded 4,695 records, and an additional 88 records were identified through targeted searches of clinical trial registries (ClinicalTrials.gov, n = 79; ICTRP, n = 9), for a total of 4,783 records. After removal of 1,577 duplicates, 3,206 unique records remained. We screened these titles and abstracts and assessed 75 full-text articles for eligibility. A total of 43 studies were excluded for wrong population (n = 25), wrong intervention (n = 10), duplicate population (n = 2), non-English (n = 2), or not addressing ankle fracture management (n = 4). Ultimately, 32 articles met the inclusion criteria for this scoping review.

### Characteristics of sources of evidence

Of the 32 studies analyzed, 24 were conducted in Europe [3, 5, 7, 8, 16–36], 5 in the USA [37–40], and 1 each in China [6], Korea [41], and New Zealand [19]. Study designs were predominantly retrospective observational (27 studies) [3, 5–8, 16–20, 23–25, 27, 29, 30, 33–37, 39–41], with 3 randomized controlled trials (RCT) [21, 31, 32], 1 prospective case series [22] and 1 ambispective cohort study [28]. Thirteen studies examined fixation methods [6, 16, 18, 19, 21, 24, 26, 28, 30–32, 35, 41], 9 investigated outcomes and complications [5, 7, 8, 29, 33, 36–38, 42], 4 addressed weight-bearing strategies [17, 22, 25, 42], 3 evaluated preoperative assessments [5, 20, 27], and 3 focused on mortality [3, 38, 39]. (Table 3) summarizes the characteristics of the included studies.

**Table 3:** Study and participant characteristics

Authors (year)	Country	Research type	Participants	N (M/F)	Age	Interventions	Outcomes
Schermann [3]	Israel	Retrospective case–control study	surgically treated for unstable ankle fractures, > 65	1045 (289/756)	NR	Open VS Closed fracture	I
Welzel [8]	Germany	Retrospective cohort study	underwent surgery, > 65	165 (34/131)	76.4	Surgical	C
Kleinsmith [37]	USA	Retrospective cohort study	nonoperative management, > 65	158 (41/117)	73.6	Nonoperative	K, L, B, C
Lin [6]	China	Retrospective case–control study	surgically treated for unstable ankle fractures, > 65	157 (102/55)	71.1	FINF VS ORIF	C, A, H
Balziano [16]	Israel	Retrospective comparative study	surgically treated for unstable ankle fractures, > 75	46 (7/39)	84.5	TTC VS ORIF	C, A
Barlow [17]	UK	Retrospective cohort study	unstable ankle fracture, > 65	161 (30/131)	80.3	EWB	K, L, C
Cay [18]	UK	Retrospective cohort study	surgically treated for open ankle fractures, > 65	34 (3/31)	87	TTC + primary wound closure	C, E, I, J
Ou [19]	New Zealand	Retrospective case–control study	surgically treated displaced ankle fractures, > 65	52 (6/46)	83.5	TTC VS ORIF	I, C, J
Aktı [20]	Turkey	Retrospective cohort study	surgically or non-surgically treatment for ankle fracture, > 65	94 (58/36)	74.54	mFI-5 predictor	C
Cho [41]	Korea	Retrospective cohort study	surgically treated for an unstable ankle fracture with medial malleolar involvement, > 65	258 (124/134)	75.7	Hook plate VS screw	B, A, C
Zyskowski [21]	Germany	RCT	surgically treated for unstable ankle fractures, > 65	39 (15/24)	77	FINF VS ORIF	C, A
Lorente [22]	Spain	Prospective multicenter cohort study	nondisplaced pronation rotation type III ankle fracture, > 65	62 (28/34)	82.9	EWB VS NWB	N, C
Spek [7]	Netherlands	Retrospective cohort study	surgically treated for ankle fractures, > 65	282 (59/223)	74.2	ORIF	C, E, I
Dang [5]	Netherlands	Retrospective cohort study	surgically treated for an LH-SER4 fracture, > 70	97 (26/71)	78.27	Investigate risk factors for LOS	D, C
Sahin [23]	Turkey	Retrospective cohort study	surgically treated for ankle fractures, > 65	111 (38/73)	70.52	Surgical	C, E, A
Tas [24]	Netherlands	Retrospective cohort study	surgically treated for Weber B ankle fractures, > 65	58 (14/44)	73.9	FINF VS ORIF	C, D
Lorente [25]	Spain	Prospective multicentre cohort	non-operative treatment for non-displaced ankle fractures, > 80	70 (31/39)	82.6	EWB VS NWB	A
Gil [38]	USA	Retrospective cohort study	The American College of Surgeons National Surgical Quality Improvement Program (ACS-NSQIP) database, > 65	2353	NR	Age > 80 VS 65–79	I, E, C
Chang [42]	USA	Retrospective cohort study	surgically treated for ankle fractures, > 65	34 (8/26)	73.4	Protocol: fixation + IWBAT	C
Aigner R (2019) [26]	Germany	Retrospective case- control study	surgically treated for ankle fractures, > 65	333 (96/237)	73.4	Locking plates VS tubular plates	C, E
Aigner [27]	Germany	Retrospective cohort study	surgically treated for ankle fractures, > 65	168 (44/124)	74.2	PDA protocol	C
Peeperkorn [28]	Belgium	Ambispective cohort study	surgically treated for AO-44B ankle fractures, > 65	112 (34/78)	73.3	FINF VS ORIF	F, A
Schray [29]	Germany	Retrospective cohort study	surgically treated for ankle fractures, > 70	58 (9/49)	77.7	Surgical	A, C, I
Herrera-Pérez [30]	Spain	Retrospective cohort study	surgically treated for ankle fractures, > 65	62 (17/45)	72.3	Locking VS Non-locking plate	C, A, J
Georgiannos [31]	Greece	RCT	closed ankle fractures & fracture-dislocations of the ankle joint, > 70	87 (23/64)	77.5	TTC nailing VS ORIF	A, B, C, D, E, J
White [32]	UK	RCT	unstable ankle fracture, > 65	100 (25/75)	74	FINF VS ORIF	A, C, F
Aigner [33]	Germany	Retrospective cohort study	surgically treated for ankle fractures, > 65	237 (80/157)	72.5	ORIF	C, E
Hsu [39]	USA	Retrospective cohort study	Centers for Medicare and Medicaid Services, > 65	19,648 (4244/15404)	77.5	Ankle fractures VS hip fractures	I, J, C
Ehrenfreund [34]	Croatia	Retrospective observational study	Weber B2 or Weber B3 ankle fracture, > 65	60	NR	surgical	A, G, C
Little [40]	USA	Retrospective case control study	surgically treated for supination external rotation (SER) ankle fractures, > 65	27 (10/17)	74	> 65 VS < 65	A, H, C
Rajeev [35]	UK	Retrospective cohort study	surgically treated for ankle fractures, > 65	24 (2/22)	79	FINF	C, A
Shivarathre [36]	UK	Retrospective cohort study	surgically treated for ankle fractures, > 80	92 (12/80)	85.2	Surgical	A, C, L

*Outcomes: A = Functional outcomes; B = Radiological outcomes; C = Complications; D = Length of stay; E = Reoperation; F = Cost-effectiveness; G = Pain (VAS); H = Reduction accuracy; I = Mortality; J = Mobility; K = Care requirements; L = Return to primary residence; M = Quality of life

ORIF = Open Reduction and Internal Fixation; TTC = Tibiotalocalcaneal nailing; FINF = Fibula Intramedullary Nail Fixation; EWB = Early Weight Bearing; NWB = Non-Weight Bearing; IWBAT = Immediate Weight Bearing As Tolerated; PVD = Peripheral Vascular Disease; mFI-5 = 5-item Modified Frailty Index; NR = Not reported.

A total of 26,284 patients were included in the 32 studies, with an average age of 77.03 years. Among them, 31 studies reported gender distribution, with 18,369 females and 5,509 males, resulting in a female-to-male ratio of 3.33:1. Additionally, eight studies described the mechanisms of injury, with low-energy trauma, such as fall from standing height or twist, accounting for the majority as 86.18% (1,353/1,570).

Four classification systems were utilized across the included studies. The malleolar classification identified bimalleolar and trimalleolar fractures as the most common types. Both the Weber and AO/OTA classification systems showed a predominance of type B fractures, while the Lauge-Hansen system indicated that supination-external rotation (SER) was the most frequent injury pattern. These findings underscore the importance of fracture classification in guiding treatment strategies, especially for unstable fractures that may require surgical fixation. Open ankle fractures were reported in only a few studies, with a combined total of 290 cases. Schermann et al. (2024)[3] conducted a retrospective case–control study comparing 128 patients with open ankle fractures to 917 patients with closed ankle fractures. Over a mean follow-up period of 4.5 years, the overall mortality rate was 27.3% in the open fracture group, compared to 15.3% in the closed fracture group (p = 0.001). These findings indicate that open ankle fractures in elderly individuals not only result in higher complication incidence but also significantly increase the risk of mortality.

### Narrative synthesis of relevant findings from the evidence

#### Clinical outcomes and complications

**Fig. 2:**
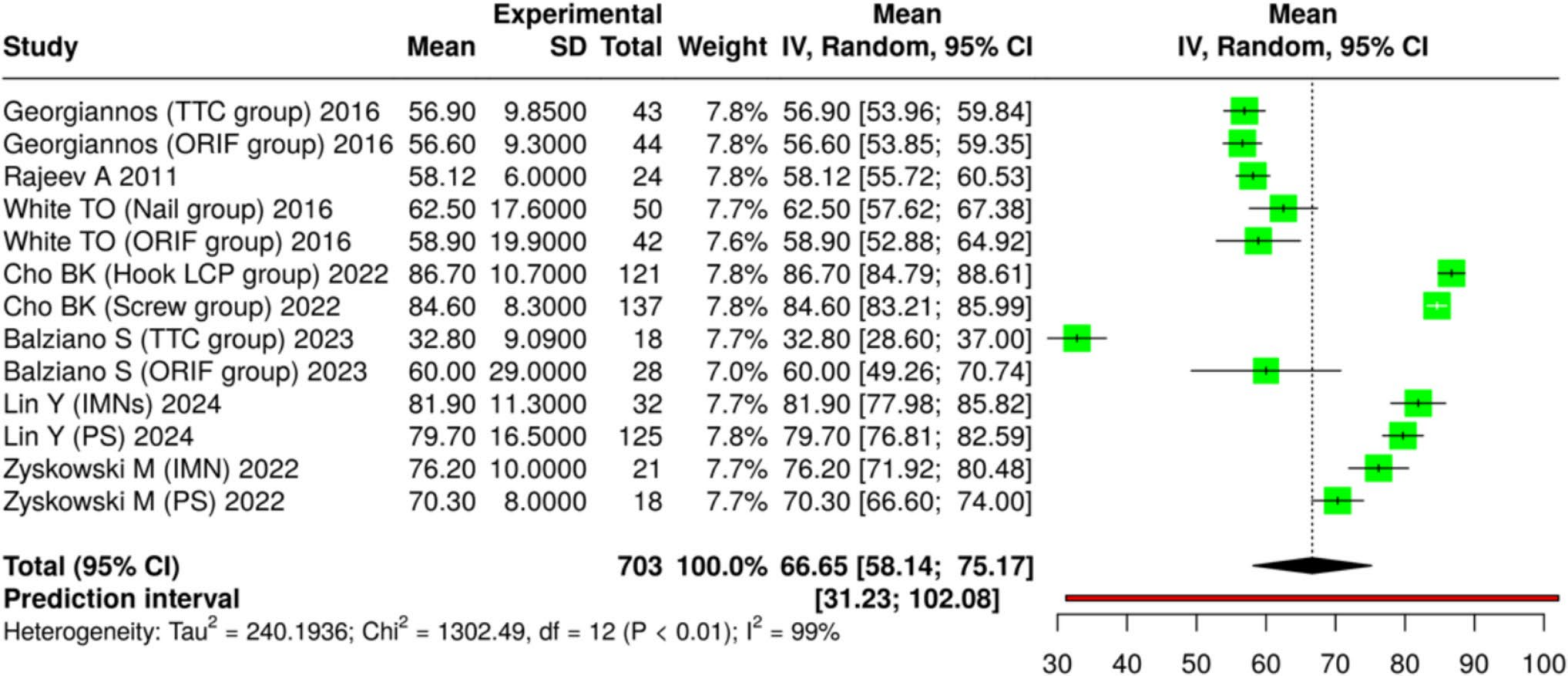
Pooled mean post-operative OMAS

**Fig. 3:**
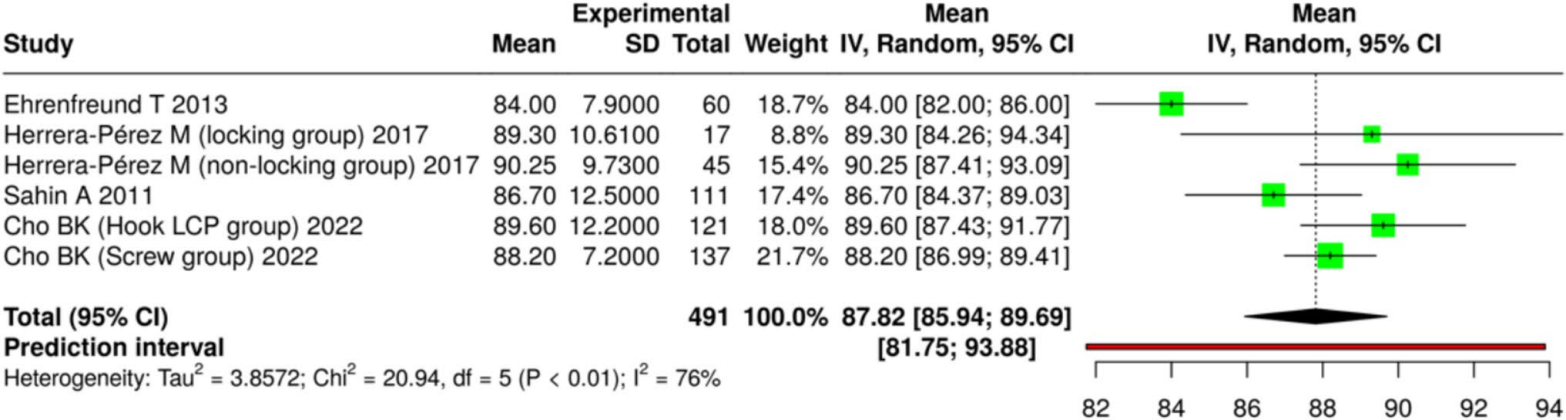
Pooled mean post-operative AOFAS

Of the 32 included studies, 11 specifically evaluated functional outcomes. The Olerud-Molander Ankle Score (OMAS) was reported in 7 studies [6, 16, 21, 31, 32, 35, 41], and the American Orthopaedic Foot and Ankle Society Ankle-Hindfoot Score (AOFAS) in 5 studies [23, 28, 30, 34, 41]. The SF-12 and Barthel index were each reported in 2 studies [22, 25]. The pooled mean postoperative OMAS was 66.65 (95% CI: 58.14–75.17; I^2^ = 99%) after a mean follow-up of 31.1 months, while the pooled mean postoperative AOFAS was 87.82 (95% CI: 85.94–89.69; I^2^ = 76%) after a mean follow-up of 35.6 months (Figs. 2 and 3).

Across eight comparative studies evaluating intramedullary nail fixation versus plate fixation, no statistically significant differences were observed in OMAS or AOFAS between groups (Table 4).

**Table 4:** Summary of functional outcomes

Number of Studies Reporting	Intervention(s)	Mean Follow-up (months)	Mean Post-op OMAS (95% CI)	Mean Post-op AOFAS (95% CI)	I^2^ (%)
7	Various surgical fixation	31.1	66.65 (58.14–75.17)	–	99
5	Various surgical fixation	35.6	–	87.82 (85.94–89.69)	76
8 comparative studies	Intramedullary nail vs plate	36.3	No significant difference	No significant difference	–

**Fig. 4:**
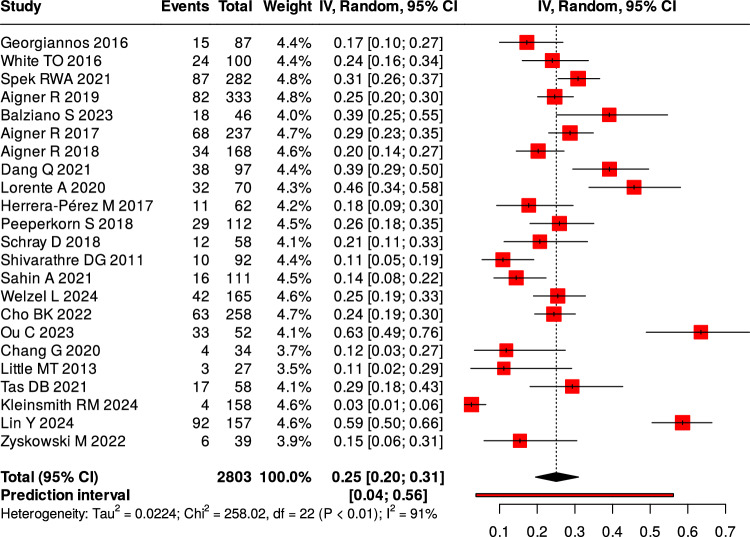
Overall proportion of complications

**Fig. 5:**
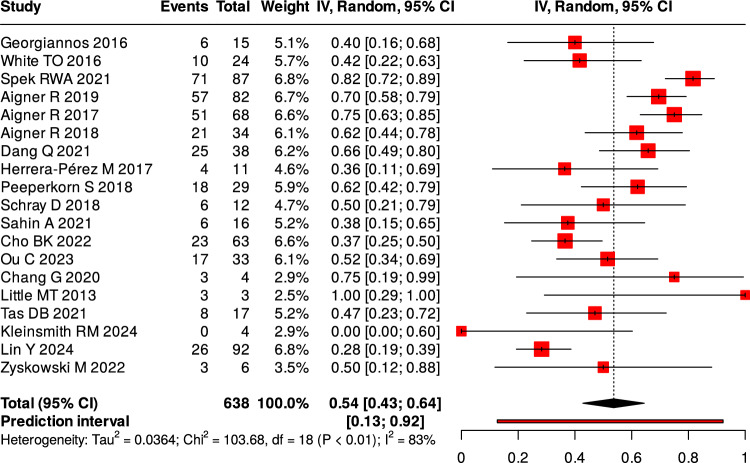
Proportion of wound-related complications among all complications

Twenty-three studies reported complications following surgical intervention. The pooled overall complication rate was of 25%, (Fig. 4). Wound-related problems—including superficial and deep infections, dehiscence, and delayed healing—accounted for 54% of all complications, (Fig. 5). Four studies provided a breakdown of wound complications, showing a deep-to-superficial ratio of approximately 1.5:1 [5, 7, 19, 41].

**Fig. 6:**
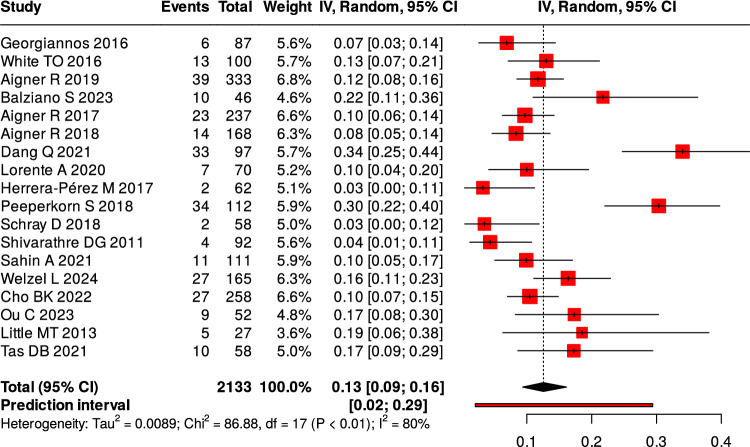
Proportion of reoperation

Reoperation occurred in 13% of patients, most often due to deep infection, fixation failure, or symptomatic implants, (Fig. 6). Nonunion was more common after nonoperative management (13.8–16.1%) than after surgery (1.9–5.8%), (Table 5).

**Table 5:** Summary of complications by category

Complication Type	Number of Studies Reporting	Pooled Frequency (%) (95% CI)	I^2^ (%)
Overall complications	23	25.0 (20.0–31.0)	91
Proportion of wound-related complications (%)	19	54.0 (43.0–64.0)	83
Reoperation	18	13.0 (9.0–16.0)	80
Nonunion (operative)	3	1.9–5.8	–
Nonunion (non-operative)	2	13.8–16.1	–

All three pooled outcomes—complication rate (I^2^ = 91%), OMAS score (I^2^ = 99%), and AOFAS score (I^2^ = 76%) — showed high heterogeneity. This reflects differences in study design, patient characteristics, fracture patterns, surgical techniques, and follow-up times. These pooled values are descriptive and should be interpreted with caution. In most studies, OMAS scores represented postoperative outcomes of different surgical fixation methods, with no direct comparisons to non-operative treatment.

#### Fixation techniques

Although both surgical and non-surgical options are available for managing ankle fractures in older adults, recent reports indicate an increasing preference for surgical management. This trend may reflect the higher proportion of unstable or complex fracture patterns in this population, which are less amenable to non-operative treatment. Regarding surgical management of a geriatric ankle fracture, several fixation strategies have been described, with varying implications for functional recovery, complication rates, and the ability to allow early weight-bearing.

To facilitate direct comparison across studies, we summarised key characteristics and findings from ten studies that compared different fixation techniques, including open reduction and internal fixation (ORIF) versus tibiotalocalcaneal (TTC) nailing, fibular intramedullary nail fixation (FINF) versus ORIF, and locking plates versus non-locking plates (Table 6).

**Table 6:** Fixation techniques comparison

Comparison	Study (Year)	Design	Main outcomes	Key findings
ORIF vs. TTC nailing	Georgiannos et al. (2017)	Retrospective cohort	Complications, reoperation, mortality	TTC had lower wound complication rate, earlier mobilization compared with ORIF
	Balziano et al. (2023)	Retrospective cohort	Revision rate, complications	TTC group had lower revision rate and fewer wound issues
	Ou & Baker (2023)	Case–control	Complications, reoperation	TTC associated with higher complication rate and reoperation rates than ORIF; most reoperations due to implant-related issues
FINF vs. ORIF	White et al. (2016)	RCT	AOFAS, wound complications, weight-bearing	No difference in AOFAS; FINF had significantly fewer wound complications and enabled earlier WB
	Zyskowski et al. (2022)	RCT	OMAS, complications	Similar OMAS; FINF had lower wound complication rate
	Tas et al. (2022)	Retrospective	Wound complications, implant failure	FINF reduced wound issues but higher implant failure in unstable fractures
	Peeperkorn et al. (2018)	Retrospective	Surgical time, wound complications	FINF had shorter operative time and fewer wound issues
	Lin et al. (2024)	Retrospective	Revision, infection, function	No significant difference in function; FINF reduced infections and reoperations in selected patients
Locking Plates vs. Non-locking Plates	Aigner et al. (2019)	Retrospective,	AOFAS, implant failure, revision rates	No significant difference in AOFAS; higher implant failure in locking group; revision rate higher with locking plates
	Herrera-Pérez et al. (2017)	Retrospective,	AOFAS, union time, wound complications	locking group had shorter partial WB time (4.7 vs. 7.8 weeks, p = 0.03)

Across studies, ORIF remains the most widely used standard approach. TTC nailing was associated with lower wound complication rates and the possibility of early weight-bearing in some studies, but findings regarding its overall complication rate were inconsistent. FINF demonstrated comparable functional outcomes to ORIF, with reduced soft-tissue complications and shorter surgical time, although biomechanical stability concerns remain, particularly for unstable fracture patterns. Locking plates provided enhanced fixation in osteoporotic bone but were associated with higher revision rates than non-locking plates. Overall, no single fixation method was clearly superior; surgical decision-making should be tailored to fracture type, bone quality, patient comorbidities, and functional demands.

#### Mortality

Mortality after geriatric ankle fractures ranged from 10 to 27.3%, with higher rates consistently observed in patients receiving nonoperative treatment, sustaining open fractures, residing in nursing facilities, or presenting with a high comorbidity burden. Short-term excess mortality in the very elderly appears largely attributable to comorbid conditions rather than age alone, underscoring the importance of preoperative risk stratification and targeted optimization strategies. Details of mortality rates and associated risk factors are summarised in (Table 7).

**Table 7:** Summary of mortality rates

Study	Cohort/intervention	Patients (n)	Follow-up	Mortality (%)	Key findings
Schermann et al., 2024	Open vs. closed fracture	187	12 mo	27.3 vs. 15.3	Open fracture independently associated with higher mortality
Hsu et al.,	Surgical vs. nonoperative	19,648	12 mo	9.1 vs. 21.5	Nonoperative care linked to increased mortality
Kadakia et al., 2015	Nursing home vs. community	257	12 mo	15.4 vs. 6.9	Institutional residence increased mortality risk
Gil et al., 2020	ORIF, 80–89 vs. 65–79 yrs	986	30 d	1.47 vs. 0.48	Higher unadjusted mortality in older group, but NS after ASA adjustment

#### Weight bearing

Four studies investigated weight-bearing strategies in patients aged 65 years and older with ankle fractures, including both surgical and non-surgical cohorts (Table 8). Overall, when appropriately selected, early weight bearing (EWB) improved functional recovery and quality of life without significantly increasing complication rates.

**Table 8:** Summary of studies on early weight bearing

Study	Study Design	Intervention / Control	Population	Main Outcomes	Key Findings
Lorente et al., 2019	Prospective cohort	EWB vs. delayed WB	Stable pronation rotation type III fractures (non-surgical)	SF-12, Barthel index	EWB improved QoL and function
Lorente et al., 2020	Prospective cohort	EWB vs. delayed WB	Stable bimalleolar fractures (non-surgical)	SF-12, Barthel index	Similar benefits without increased complications
Chang et al., 2020	Retrospective	IWBAT vs. NWB	Surgically treated	Complications, discharge disposition	IWBAT did not increase complications; higher rate of home discharge
Barlow et al., 2023	Retrospective	FINF (earlier WB) vs. other fixation	Surgically treated	Time to WB, discharge outcomes	FINF group started WB ~ 3 weeks earlier; higher home discharge rate

The available evidence supports the potential benefits of EWB in reducing dependency and enhancing recovery in frail older adults with ankle fractures. However, weight-bearing protocols should be individualized, taking into account fracture stability, fixation method, and comorbidities to balance functional gains against complication risks.

#### Patient evaluation

**Fig. 7:**
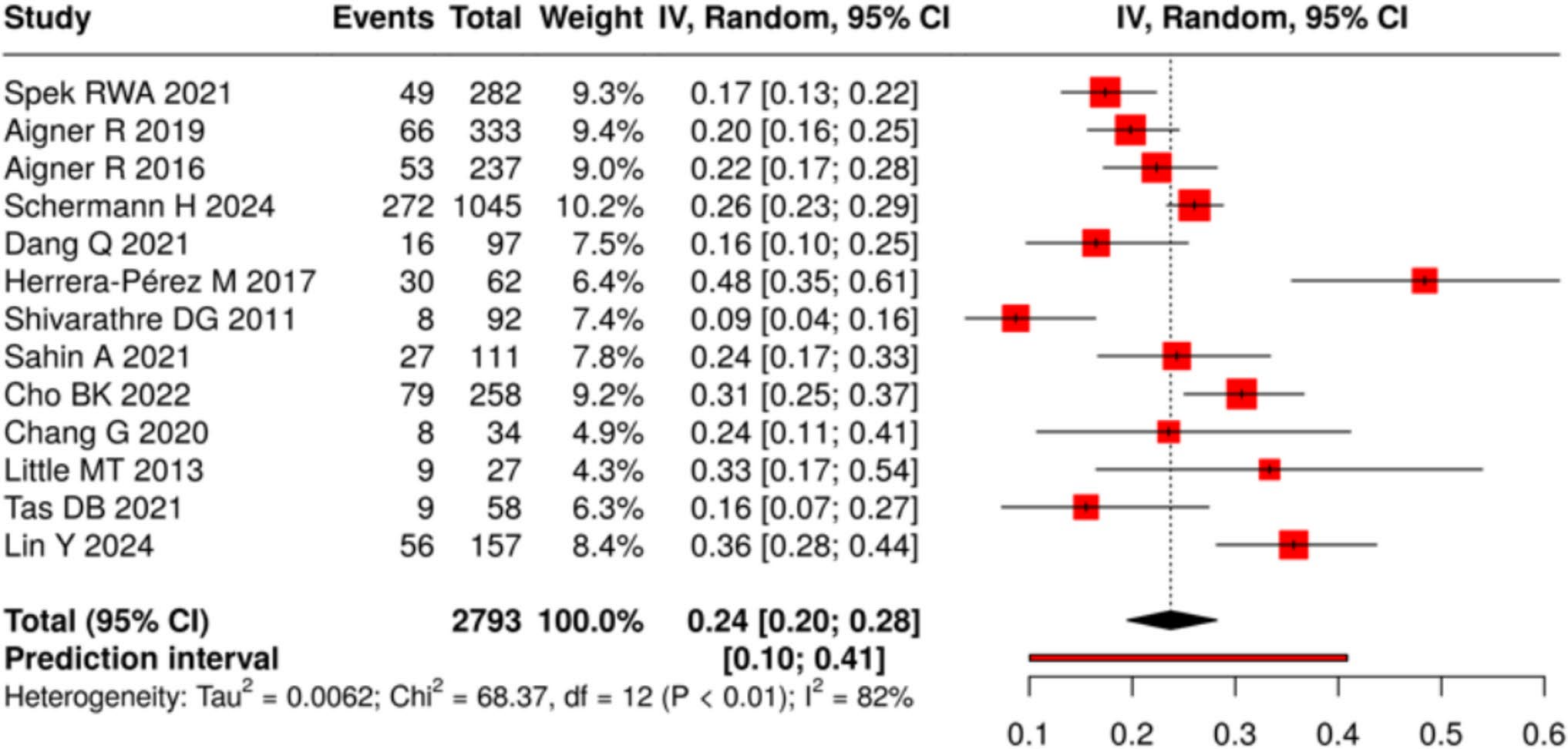
Prevalence of diabetes across included cohorts

**Fig. 8:**
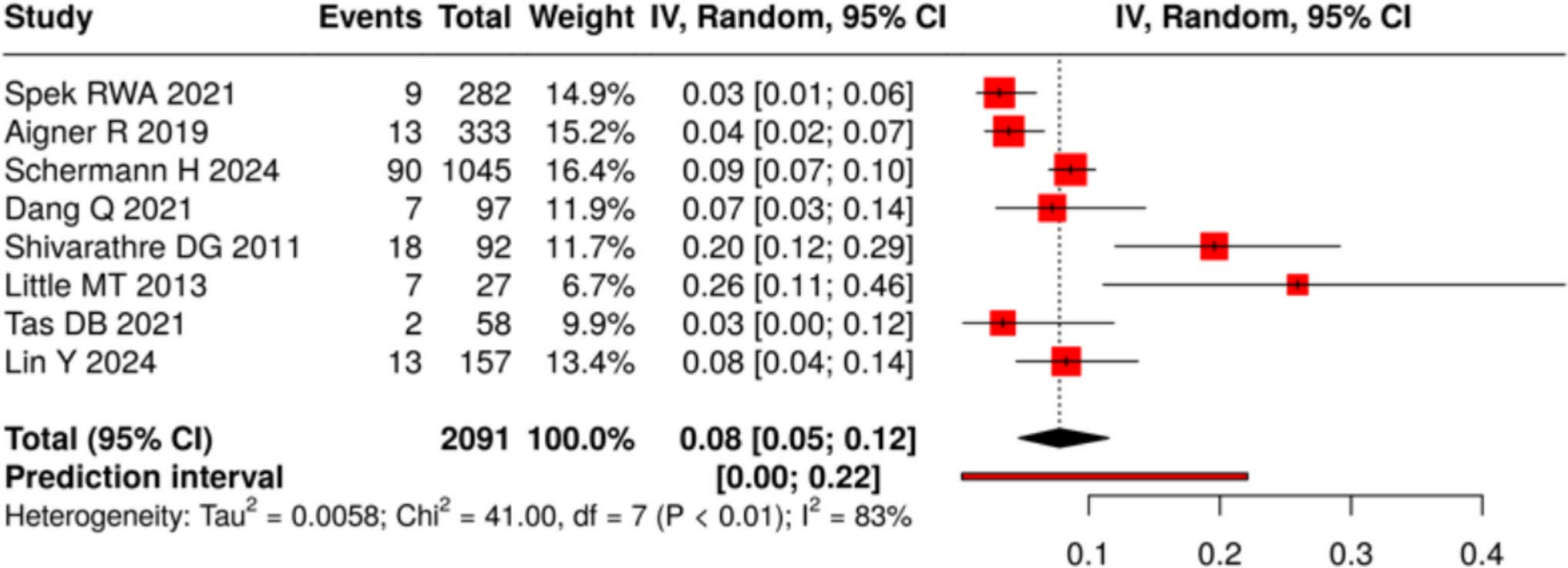
Prevalence of PVD across included cohorts

Preoperative assessment is critical in elderly ankle fracture patients to identify modifiable risks and guide surgical planning. Common factors such as diabetes, peripheral vascular disease (PVD), and frailty substantially increase postoperative complication rates. Across included studies, the pooled prevalence of diabetes was 24% (95% CI: 20–28%; I^2^ = 82%), ranging from 9.0 to 36%. The pooled prevalence of PVD was 8% (95% CI: 5–12%; I^2^ = 83%), (Figs. 7 and 8).Evidence supports the use of standardized vascular screening and frailty indices to better stratify risk and target optimization measures. Comorbidities and frailty are prevalent in this population and strongly influence outcomes. Structured preoperative screening—particularly vascular assessment and frailty scoring—can help reduce complications and improve recovery (Table 9).

**Table 9:** Summary of comorbidity and risk assessment findings

Study	Risk factor / tool	Prevalence / OR (95% CI)	Key findings
Multiple studies	Diabetes	24% (20–28%); range 9–36%	Higher rates of wound complications; significant heterogeneity
Multiple studies	PVD	8% (5–12%)	Standardized vascular assessment reduced wound complication rates
Aktı et al., 2020	mFI-5 frailty index	OR = 3.4 (readmission); OR = 2.86 (wound infection); OR = 5.16 (life-threatening complications)	Outperformed conventional risk tools in predicting adverse events
Welzel et al., 2018	Comorbidity burden	Overall complication rate 26% in elderly vs. 8% in younger patients (p < 0.001)	Delaying surgery for optimization reduced major complications from 90 to 30%

## Discussion

This scoping review systematically identified and synthesized the available literature on the management of ankle fractures in older adults. A total of 32 studies were included, the majority being retrospective observational designs conducted in Europe and North America. The mean patient age across studies exceeded 77 years, with a consistent female predominance reflecting the osteoporosis-related nature of these injuries. The evidence base demonstrated marked heterogeneity, particularly in treatment strategies, fixation techniques, weight-bearing protocols, and perioperative risk assessment. While surgical fixation remains the most frequently reported approach, no single technique has achieved universal consensus. Importantly, the predominance of retrospective data, limited prospective trials, and variability in outcome reporting restrict the strength and generalizability of conclusions.

The following sections expand on key themes identified in the literature, including clinical outcomes, fixation methods, rehabilitation strategies, comorbidity impact, and mortality.

### Clinical outcomes and complications

#### Clinical outcomes

Across the included studies, postoperative functional assessments—most commonly assessed with OMAS and the AOFAS Score—generally indicated satisfactory recovery following surgical management[6, 16, 21, 30–32, 34, 35, 41]. However, results varied considerably depending on comorbidities, fracture characteristics, and rehabilitation strategies. Patients with severe osteoporosis, multiple comorbidities, or complex fracture patterns were more likely to experience prolonged impairment and limited return to baseline mobility [22]. Reported postoperative OMAS scores for different surgical fixation methods were generally in the moderate-to-good range, and AOFAS scores frequently exceeded 80 points, indicating good-to-excellent results according to commonly used cut-offs in previous literature. However, these cut-offs are not part of the original scoring systems and vary between studies. Moreover, the absence of direct comparisons with conservative management means these results should be interpreted within the surgical context only.

Functional recovery is further influenced by weight-bearing protocols and rehabilitation strategies. Several studies suggested that structured rehabilitation programs emphasizing early mobilization can enhance functional outcomes and reduce dependence on assistive devices [17, 42]. Nevertheless, recommendations remain inconsistent, with protocols often differing according to fixation method and patient-specific factors. Some studies reported that delayed weight-bearing in osteoporotic patients resulted in deconditioning and prolonged rehabilitation, underscoring the importance of individualized planning [43]. Patient-reported outcomes also indicated that, although most older adults regained satisfactory function, persistent pain, stiffness, and reduced mobility were common compared with pre-injury levels [37]. This was especially evident in patients with preexisting musculoskeletal conditions, such as osteoarthritis or PVD, which further compromised recovery [27].

Beyond physical outcomes, the psychosocial impact of geriatric ankle fractures should not be overlooked. Reduced mobility is strongly associated with loss of independence and increased risk of institutionalization [8]. Considering these challenges, future research should aim to develop rehabilitation programs tailored to frail older adults, focusing not only on radiographic healing and complication rates but also on meaningful outcomes such as independent living, walking ability, and quality of life. More robust prospective studies with standardized outcome measures are needed to better define the long-term functional trajectory of this population and to support evidence-based decision-making [8, 22, 27, 37].

### Complications

Postoperative complications remain a major concern in elderly ankle fracture management. The fragile soft-tissue envelope, reduced peripheral circulation, and osteoporotic bone quality in frail older adults increase the risks of wound dehiscence, fixation failure, and delayed union, underscoring the need for tailored surgical and postoperative strategies. Wound-related complications were the most frequent, accounting for more than half of reported postoperative events. Deep infections occurred more often than superficial ones [5, 7, 19, 41]. These findings highlight the vulnerability of this population to severe soft-tissue problems, which often lead to prolonged recovery and functional decline. Future approaches should therefore emphasize minimally invasive fixation and advanced wound care protocols.

Several comorbidities—particularly diabetes, PVD, and frailty—further amplify the risks of wound breakdown, infection, and impaired healing. To address these challenges, many studies recommended preoperative risk stratification using frailty indices and vascular assessments to identify high-risk patients and guide treatment decisions. Minimally invasive fixation methods, such as FINF and TTC nailing, were reported to reduce soft-tissue complications while maintaining function, two studies comparing TTC nailing and ORIF, and another two comparing FINF and ORIF, reported similar AOFAS scores between groups, indicating comparable short-term functional outcomes, although further research is needed to confirm their long-term stability in osteoporotic bone.

Open fractures represent a particularly high-risk subgroup, with substantially higher infection, delayed healing, and mortality rates compared with closed injuries [3]. The compromised soft-tissue integrity and vascular supply in open fractures contribute to increased complications, requiring early debridement, infection control, and soft-tissue reconstruction. In severe situations with extensive ischemia, primary amputation may be required. These findings are consistent with broader trauma literature and reinforce the need for multidisciplinary care pathways combining orthopedic, vascular, and plastic surgical expertise [44, 45].

### Fixation strategies

Surgical fixation remains the standard treatment for displaced fractures, but no single technique has demonstrated clear superiority. ORIF is the most commonly applied method, though it carries a considerable risk of wound complications,, particularly in diabetic patients and those with PVD. This has driven interest in alternative strategies that may reduce soft-tissue morbidity.

Minimally invasive approaches, such as FINF and TTC nailing, are increasingly reported as viable alternatives. Evidence suggests that both techniques can lower wound-related complications and allow for early mobilization, though each presents trade-offs [46]. FINF may be less stable in severely osteoporotic or comminuted fractures, while TTC nailing sacrifices subtalar joint motion and may predispose to implant failure or adjacent joint degeneration [47]. These findings are consistent with a previous systematic review, which also supported the use of TTC nailing in elderly patients with fragility fractures, reporting low complication and mortality rates and favorable functional recovery [48]. Locking plates have also been advocated for osteoporotic bone, but concerns about higher revision rates remain [7]. Overall, these findings highlight the importance of tailoring fixation choice to fracture stability, soft-tissue condition, and patient comorbidities rather than applying a one-size-fits-all strategy.

Nonoperative treatment remains appropriate for selected patients, particularly frail older adults with stable fracture patterns or high surgical risks. While such management may limit early mobility, some studies report that long-term independence can still be preserved in carefully chosen cases.

However, standardized selection criteria and rehabilitation pathways are lacking, and further prospective studies are needed to define the optimal role of conservative management in this population [37].However, standardized selection criteria and rehabilitation pathways are lacking, and further prospective studies are needed to define the optimal role of conservative management in this population. Importantly, the choice of fixation method not only determines immediate stability but also directly influences the feasibility and timing of postoperative weight-bearing, which is a key factor in functional recovery for elderly patients.

### Weight-bearing and rehabilitation strategies

Weight-bearing (WB) protocols in geriatric ankle fractures remain heterogeneous, with two broad approaches: early weight-bearing (EWB, typically initiated around two weeks postoperatively) and delayed or non-weight-bearing (NWB, usually restricted for six weeks). Although EWB may enhance functional recovery, reduce deconditioning, and shorten hospital stay, the supporting evidence is limited and often underpowered [49]. In elderly patients, the balance becomes more complex due to osteoporosis, reduced healing capacity, and higher risk of fixation failure. Careful patient selection therefore remains essential [17].

For non-surgical management, EWB appears safe in selected cases with non-displaced or stable fractures following closed reduction. However, protocols vary widely—from immediate full weight-bearing (FWB) in protective boots to gradual progression with partial weight-bearing (PWB) [25]. Standardized criteria for conservative care are still lacking, making it difficult to balance early mobility benefits against the risk of secondary displacement.

In surgically treated patients, feasibility of EWB depends on implant stability, fracture pattern, and soft-tissue status. Certain techniques, including intramedullary fixation or stable plating constructs, have been associated with safe progression to EWB, but no universally accepted protocol exists [17]. Current practices differ in timing, ranging from immediate to delayed initiation, and in loading extent, with some advocating PWB and others supporting FWB as tolerated.

Overall, while early mobilization is increasingly recognized as critical for maintaining independence in frail older adults, evidence-based, fracture-specific WB protocols are still lacking. Future studies should prioritize standardized rehabilitation pathways that consider both fixation stability and patient frailty, aiming to optimize recovery while minimizing complications. Equally important, the success of both fixation and rehabilitation strategies is heavily modulated by patient comorbidities, which play a decisive role in surgical risk and long-term recovery.

### The impact of comorbidities on outcomes

Comorbidities, particularly diabetes and PVD, significantly influence perioperative risks and postoperative outcomes. This review found a pooled prevalence of 24% for diabetes and 8% for PVD, although variability between studies was considerable. Both conditions are strongly linked to higher rates of wound complications, infection, and delayed healing.

Aigner et al. highlighted the importance of identifying undiagnosed PVD, showing that systematic vascular assessment with noninvasive exams and CTA enabled early intervention and reduced wound problems [27]. Similarly, metabolic comorbidities such as diabetes not only impair wound healing but also complicate perioperative management.

Beyond metabolic and vascular factors, frailty remains a key determinant of postoperative complications. Aktı et al. reported that the 5-factor modified Frailty Index (mFI-5) is an effective predictor of hospital readmission, wound infection, and major medical events [20]. Similarly, Welzel et al. found that elderly patients had a 26% complication rate compared to 8% in younger individuals, reinforcing the profound impact of multimorbidity on surgical outcomes [8].

Despite evidence supporting structured preoperative screening, standardized protocols for incorporating vascular assessment, frailty scoring, and metabolic control into treatment planning remain lacking. Future research should focus on integrating individualized risk stratification models into clinical practice to balance fracture stabilization with comorbidity management.

### Mortality and long-term prognosis

Reported mortality rates after geriatric ankle fractures range from 10 to 27%, reflecting the influence of fracture severity, treatment strategy, and comorbidities. Large database studies consistently suggest higher mortality in nonoperative patients, although this may partly reflect selection bias since these individuals are often frailer and more medically complex [39, 50, 51]. Open fractures represent a particularly high-risk subgroup, with one study reporting 1-year mortality of 8% for open versus 3.2% for closed fractures, and overall mortality rates of 27.3% and 15.3%, respectively [3]. These findings imply that both the injury pattern and complications such as infection, reoperation, and wound healing failure substantially affect survival.

Functional decline and immobility further contribute to excess mortality. Gil et al. observed disproportionately high mortality among patients discharged to nursing facilities, underscoring the role of loss of independence and secondary complications such as pneumonia and thromboembolism [35, 50]. This aligns with evidence from hip fracture populations, where mobility preservation is strongly linked to improved survival.

Despite these insights, most available studies did not perform multivariable analyses, limiting causal interpretation. The impact of factors such as surgical timing, rehabilitation intensity, and comorbidity control on long-term prognosis therefore remains uncertain. Future research should develop risk-adjusted treatment pathways that balance the survival benefits of surgery with frailty-related risks, and should test perioperative optimisation strategies—including geriatric assessment, prehabilitation, and multimodal care—in prospective designs.

### Limitations

This review has several limitations. First, substantial heterogeneity was present across study designs, populations, and methodologies. Pooled estimates for complication rate (I^2^ = 91%), OMAS (I^2^ = 99%), and AOFAS (I^2^ = 76%) should therefore be interpreted as broad indicators rather than precise measures.

Second, in line with PRISMA-ScR methodology, no formal risk of bias assessment was performed, as the primary aim was to map existing evidence rather than provide definitive effect estimates. The pooled descriptive analyses and forest plots were included only as complementary tools to illustrate outcome variability, not as a full meta-analysis.

Third, most included studies were retrospective, introducing selection bias and limiting causal inference. In addition, subgroup analyses by comorbidity burden, frailty, or cognitive status were largely absent, even though these factors strongly influence complications, function, and mortality.

Finally, large database studies offer broad generalizability but lack detailed clinical insights, while single-center studies provide specificity but limited external validity. Differences in healthcare systems and inconsistent comorbidity reporting further hinder comparability. Future prospective multicenter studies with standardized outcomes, stratification by frailty and comorbidities, and longer follow-up are needed to refine evidence-based strategies for geriatric ankle fracture care.

## Conclusions

This scoping review mapped the evidence on geriatric ankle fracture management. Surgical fixation is the predominant approach, with ORIF most common and TTC nailing or FINF as alternatives, but no technique has proven clearly superior. Evidence highlights the importance of individualized strategies and multidisciplinary care. Given that most studies are retrospective and heterogeneous, findings should be interpreted as descriptive rather than definitive. Future prospective research should focus on long-term function, standardized outcome measures, and weight-bearing protocols.

## Data Availability

No datasets were generated or analysed during the current study.
